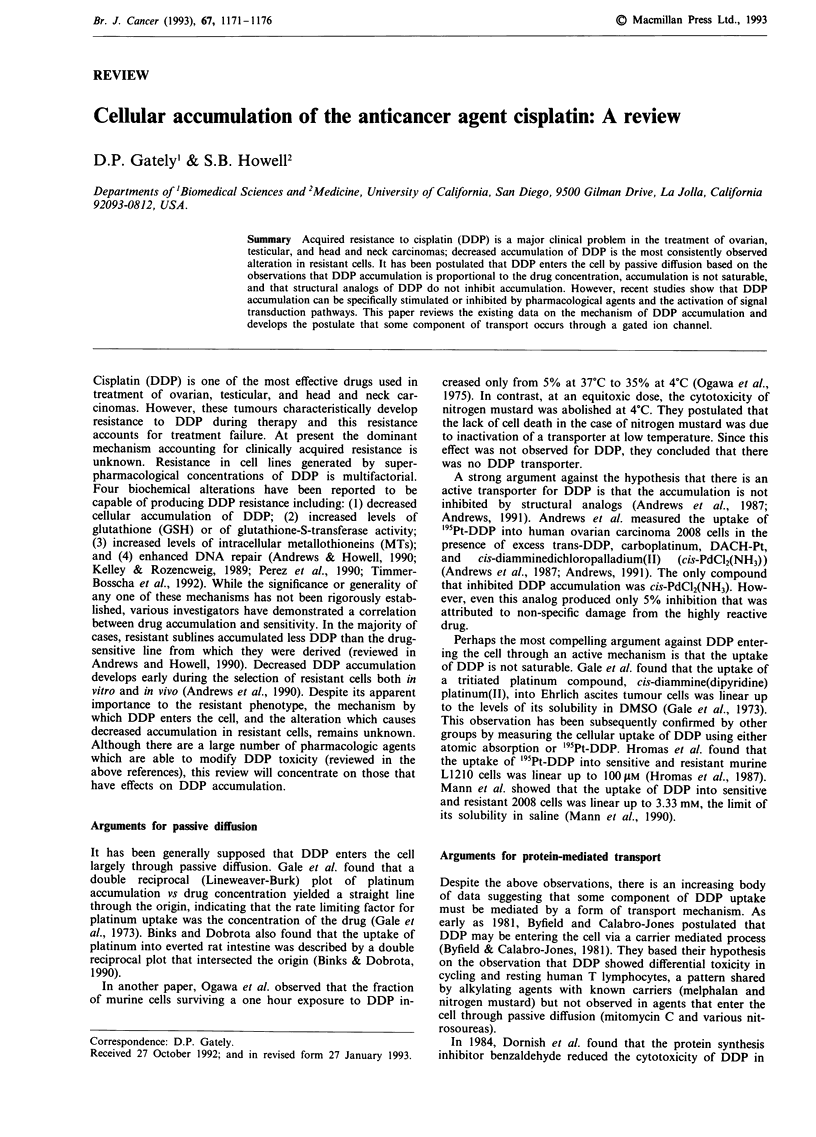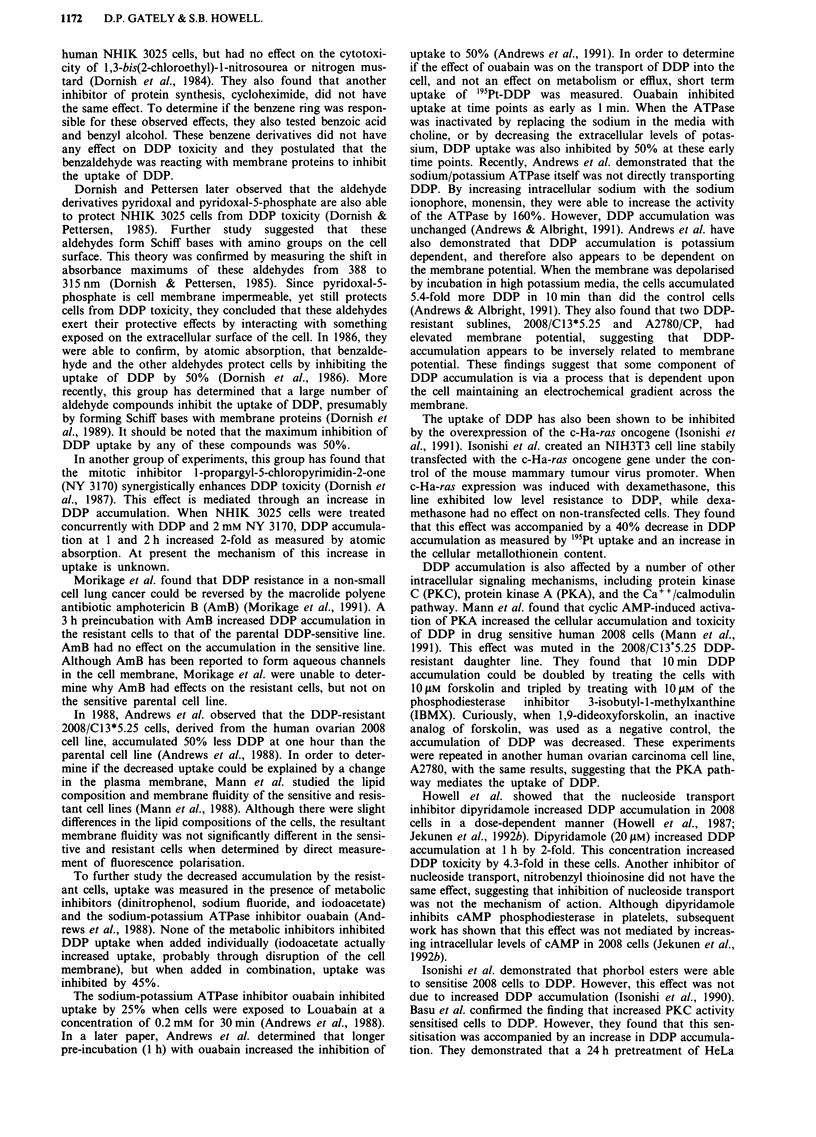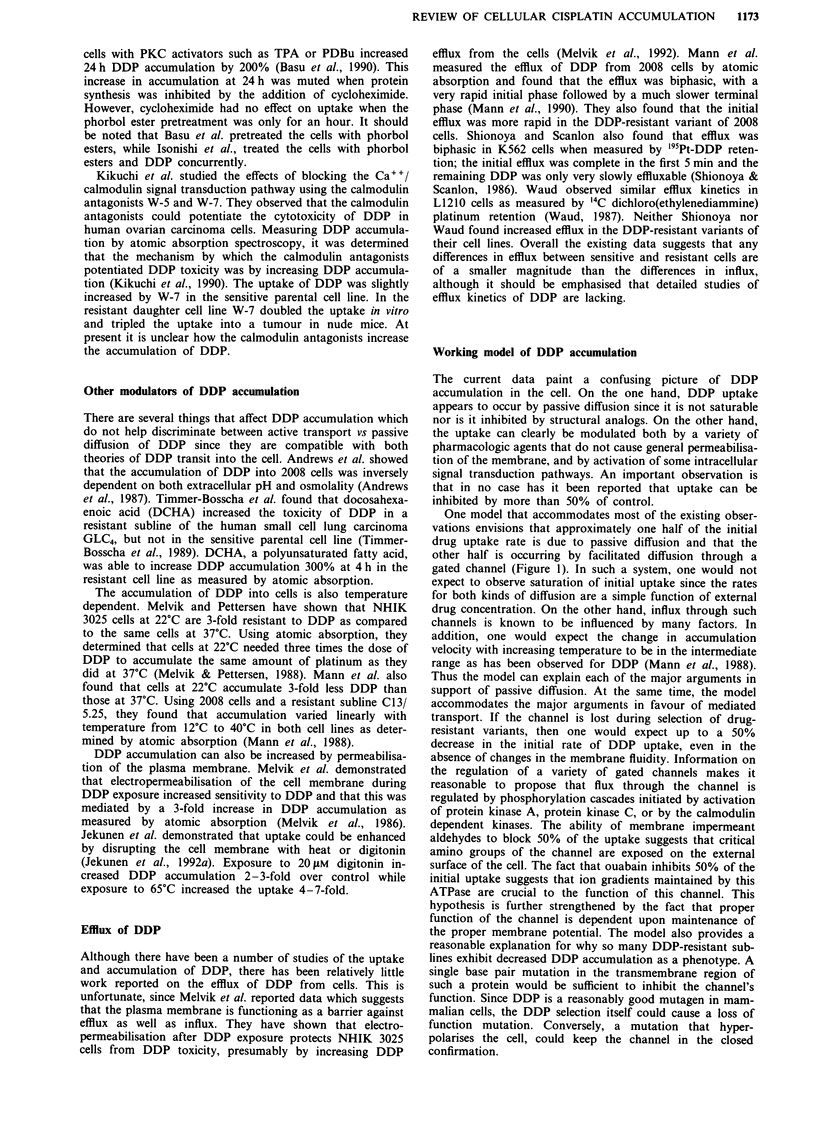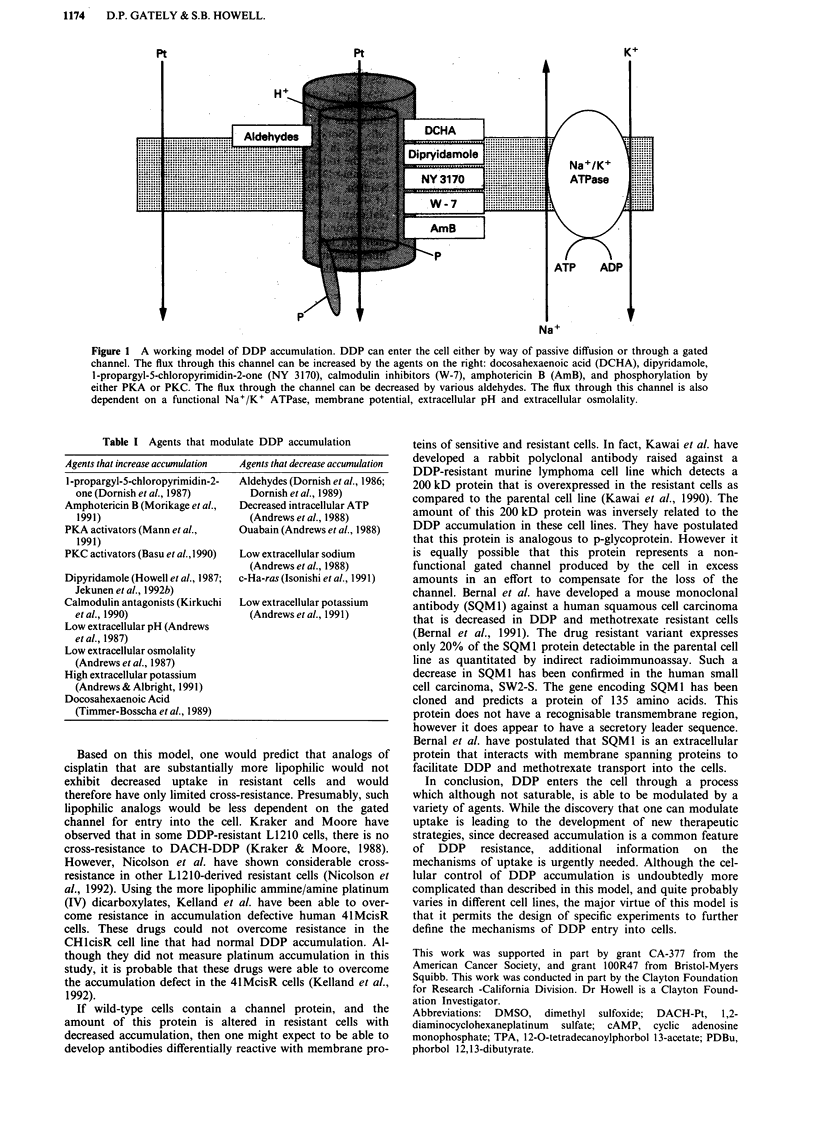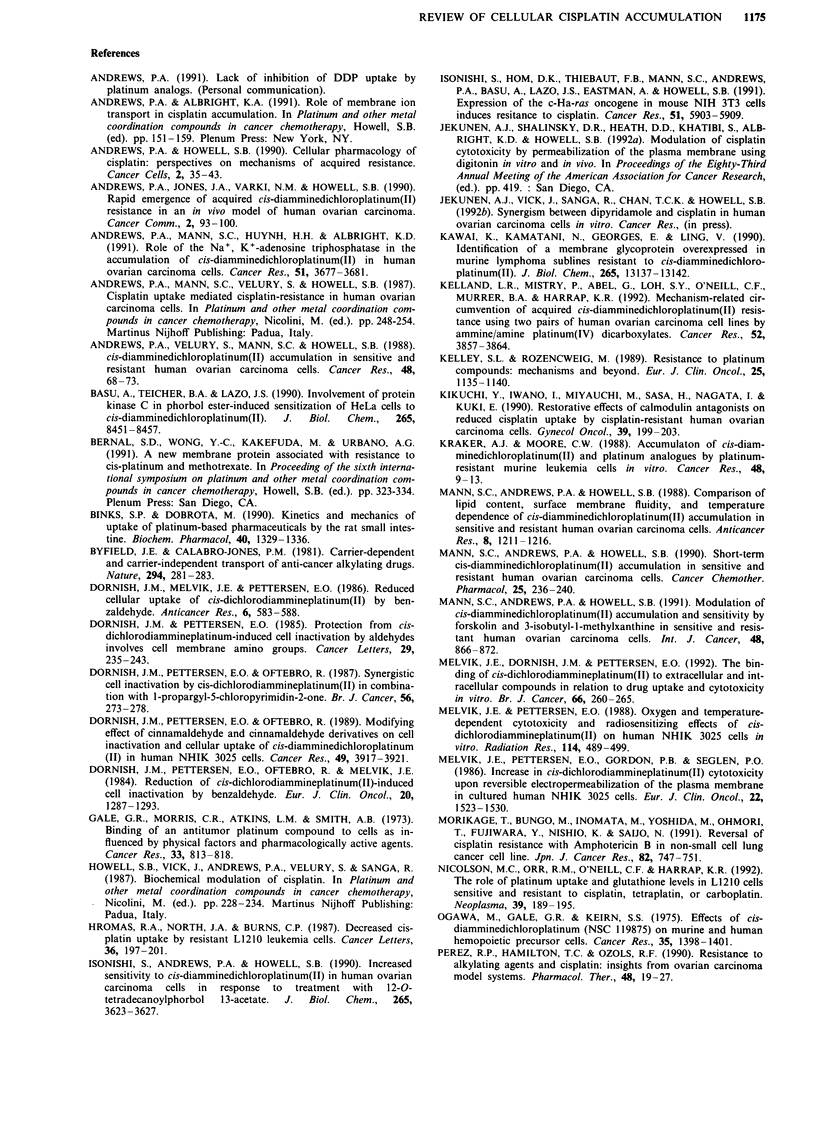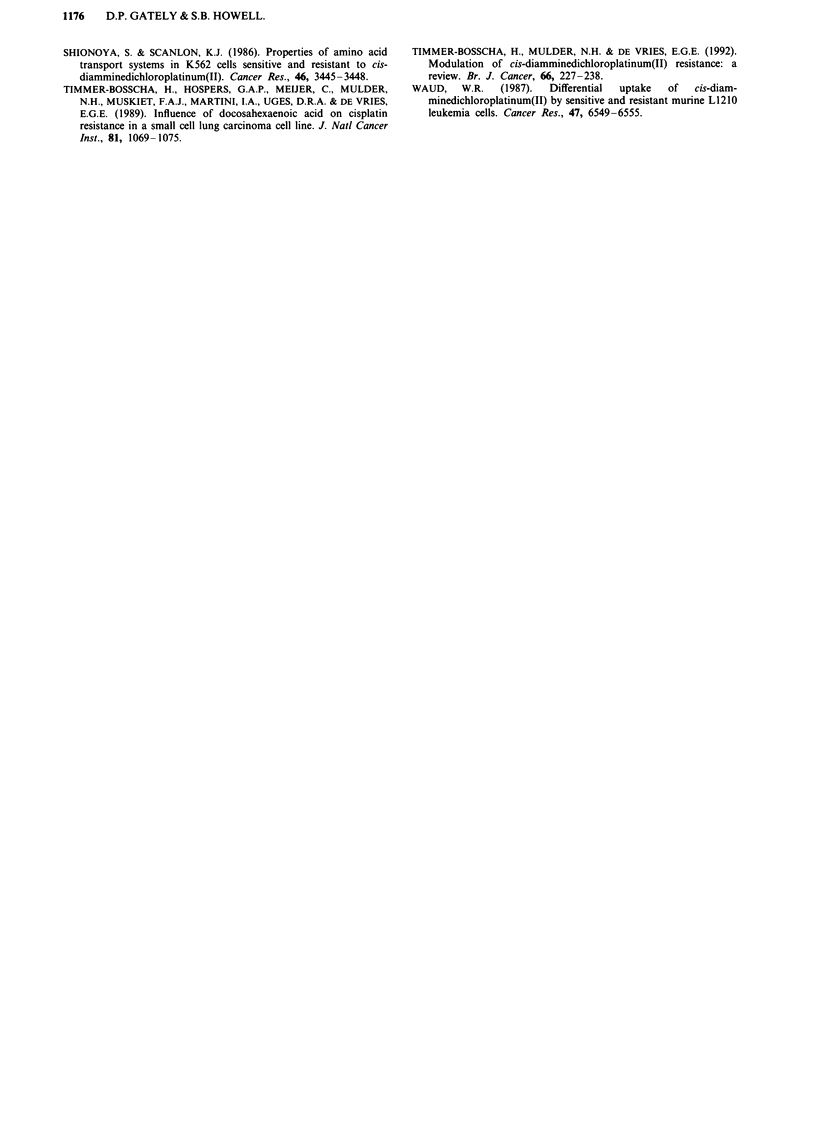# Cellular accumulation of the anticancer agent cisplatin: a review.

**DOI:** 10.1038/bjc.1993.221

**Published:** 1993-06

**Authors:** D. P. Gately, S. B. Howell

**Affiliations:** Department of Biomedical Sciences, University of California, San Diego, La Jolla 92093-0812.

## Abstract

Acquired resistance to cisplatin (DDP) is a major clinical problem in the treatment of ovarian, testicular, and head and neck carcinomas; decreased accumulation of DDP is the most consistently observed alteration in resistant cells. It has been postulated that DDP enters the cell by passive diffusion based on the observations that DDP accumulation is proportional to the drug concentration, accumulation is not saturable, and that structural analogs of DDP do not inhibit accumulation. However, recent studies show that DDP accumulation can be specifically stimulated or inhibited by pharmacological agents and the activation of signal transduction pathways. This paper reviews the existing data on the mechanism of DDP accumulation and develops the postulate that some component of transport occurs through a gated ion channel.


*Br. J. Cancer* (1993), **67**, 1171–1176 © Macmillan Press Ltd., 1993

**REVIEW**

### Cellular accumulation of the anticancer agent cisplatin: A review

D.P. Gately[1] & S.B. Howell[2]

*Departments of [1]Biomedical Sciences and [2]Medicine, University of California, San Diego, 9500 Gilman Drive, La Jolla, California 92093-0812, USA.*

**Summary** Acquired resistance to cisplatin (DDP) is a major clinical problem in the treatment of ovarian, testicular, and head and neck carcinomas; decreased accumulation of DDP is the most consistently observed alteration in resistant cells. It has been postulated that DDP enters the cell by passive diffusion based on the observations that DDP accumulation is proportional to the drug concentration, accumulation is not saturable, and that structural analogs of DDP do not inhibit accumulation. However, recent studies show that DDP accumulation can be specifically stimulated or inhibited by pharmacological agents and the activation of signal transduction pathways. This paper reviews the existing data on the mechanism of DDP accumulation and develops the postulate that some component of transport occurs through a gated ion channel.

Cisplatin (DDP) is one of the most effective drugs used in treatment of ovarian, testicular, and head and neck carcinomas. However, these tumours characteristically develop resistance to DDP during therapy and this resistance accounts for treatment failure. At present the dominant mechanism accounting for clinically acquired resistance is unknown. Resistance in cell lines generated by super-pharmacological concentrations of DDP is multifactorial. Four biochemical alterations have been reported to be capable of producing DDP resistance including: (1) decreased cellular accumulation of DDP; (2) increased levels of glutathione (GSH) or of glutathione-S-transferase activity; (3) increased levels of intracellular metallothioneins (MTs); and (4) enhanced DNA repair (Andrews & Howell, 1990; Kelley & Rozencweig, 1989; Perez *et al.*, 1990; Timmer-Bosscha *et al.*, 1992). While the significance or generality of any one of these mechanisms has not been rigorously established, various investigators have demonstrated a correlation between drug accumulation and sensitivity. In the majority of cases, resistant sublines accumulated less DDP than the drug-sensitive line from which they were derived (reviewed in Andrews and Howell, 1990). Decreased DDP accumulation develops early during the selection of resistant cells both *in vitro* and *in vivo* (Andrews *et al.*, 1990). Despite its apparent importance to the resistant phenotype, the mechanism by which DDP enters the cell, and the alteration which causes decreased accumulation in resistant cells, remains unknown. Although there are a large number of pharmacologic agents which are able to modify DDP toxicity (reviewed in the above references), this review will concentrate on those that have effects on DDP accumulation.

### Arguments for passive diffusion

It has been generally supposed that DDP enters the cell largely through passive diffusion. Gale *et al.* found that a double reciprocal (Lineweaver-Burk) plot of platinum accumulation *vs* drug concentration yielded a straight line through the origin, indicating that the rate limiting factor for platinum uptake was the concentration of the drug (Gale *et al.*, 1973). Binks and Dobrota also found that the uptake of platinum into everted rat intestine was described by a double reciprocal plot that intersected the origin (Binks & Dobrota, 1990).

In another paper, Ogawa *et al.* observed that the fraction of murine cells surviving a one hour exposure to DDP increased only from 5% at 37°C to 35% at 4°C (Ogawa *et al.*, 1975). In contrast, at an equitoxic dose, the cytotoxicity of nitrogen mustard was abolished at 4°C. They postulated that the lack of cell death in the case of nitrogen mustard was due to inactivation of a transporter at low temperature. Since this effect was not observed for DDP, they concluded that there was no DDP transporter.

A strong argument against the hypothesis that there is an active transporter for DDP is that the accumulation is not inhibited by structural analogs (Andrews *et al.*, 1987; Andrews, 1991). Andrews *et al.* measured the uptake of $^{195}$Pt-DDP into human ovarian carcinoma 2008 cells in the presence of excess trans-DDP, carboplatinum, DACH-Pt, and *cis*-diamminedichloropalladium(II) (*cis*-$PdCl_2(NH_3)$) (Andrews *et al.*, 1987; Andrews, 1991). The only compound that inhibited DDP accumulation was *cis*-$PdCl_2(NH_3)$. However, even this analog produced only 5% inhibition that was attributed to non-specific damage from the highly reactive drug.

Perhaps the most compelling argument against DDP entering the cell through an active mechanism is that the uptake of DDP is not saturable. Gale *et al.* found that the uptake of a tritiated platinum compound, *cis*-diammine(dipyridine) platinum(II), into Ehrlich ascites tumour cells was linear up to the levels of its solubility in DMSO (Gale *et al.*, 1973). This observation has been subsequently confirmed by other groups by measuring the cellular uptake of DDP using either atomic absorption or $^{195}$Pt-DDP. Hromas *et al.* found that the uptake of $^{195}$Pt-DDP into sensitive and resistant murine L1210 cells was linear up to 100 $\mu$M (Hromas *et al.*, 1987). Mann *et al.* showed that the uptake of DDP into sensitive and resistant 2008 cells was linear up to 3.33 mM, the limit of its solubility in saline (Mann *et al.*, 1990).

### Arguments for protein-mediated transport

Despite the above observations, there is an increasing body of data suggesting that some component of DDP uptake must be mediated by a form of transport mechanism. As early as 1981, Byfield and Calabro-Jones postulated that DDP may be entering the cell via a carrier mediated process (Byfield & Calabro-Jones, 1981). They based their hypothesis on the observation that DDP showed differential toxicity in cycling and resting human T lymphocytes, a pattern shared by alkylating agents with known carriers (melphalan and nitrogen mustard) but not observed in agents that enter the cell through passive diffusion (mitomycin C and various nitrosoureas).

In 1984, Dornish *et al.* found that the protein synthesis inhibitor benzaldehyde reduced the cytotoxicity of DDP in

Correspondence: D.P. Gately.
Received 27 October 1992; and in revised form 27 January 1993.

human NHIK 3025 cells, but had no effect on the cytotoxicity of 1,3-*bis*(2-chloroethyl)-1-nitrosourea or nitrogen mustard (Dornish *et al.*, 1984). They also found that another inhibitor of protein synthesis, cycloheximide, did not have the same effect. To determine if the benzene ring was responsible for these observed effects, they also tested benzoic acid and benzyl alcohol. These benzene derivatives did not have any effect on DDP toxicity and they postulated that the benzaldehyde was reacting with membrane proteins to inhibit the uptake of DDP.

Dornish and Pettersen later observed that the aldehyde derivatives pyridoxal and pyridoxal-5-phosphate are also able to protect NHIK 3025 cells from DDP toxicity (Dornish & Pettersen, 1985). Further study suggested that these aldehydes form Schiff bases with amino groups on the cell surface. This theory was confirmed by measuring the shift in absorbance maximums of these aldehydes from 388 to 315 nm (Dornish & Pettersen, 1985). Since pyridoxal-5-phosphate is cell membrane impermeable, yet still protects cells from DDP toxicity, they concluded that these aldehydes exert their protective effects by interacting with something exposed on the extracellular surface of the cell. In 1986, they were able to confirm, by atomic absorption, that benzaldehyde and the other aldehydes protect cells by inhibiting the uptake of DDP by 50% (Dornish *et al.*, 1986). More recently, this group has determined that a large number of aldehyde compounds inhibit the uptake of DDP, presumably by forming Schiff bases with membrane proteins (Dornish *et al.*, 1989). It should be noted that the maximum inhibition of DDP uptake by any of these compounds was 50%.

In another group of experiments, this group has found that the mitotic inhibitor 1-propargyl-5-chloropyrimidin-2-one (NY 3170) synergistically enhances DDP toxicity (Dornish *et al.*, 1987). This effect is mediated through an increase in DDP accumulation. When NHIK 3025 cells were treated concurrently with DDP and 2 mM NY 3170, DDP accumulation at 1 and 2 h increased 2-fold as measured by atomic absorption. At present the mechanism of this increase in uptake is unknown.

Morikage *et al.* found that DDP resistance in a non-small cell lung cancer could be reversed by the macrolide polyene antibiotic amphotericin B (AmB) (Morikage *et al.*, 1991). A 3 h preincubation with AmB increased DDP accumulation in the resistant cells to that of the parental DDP-sensitive line. AmB had no effect on the accumulation in the sensitive line. Although AmB has been reported to form aqueous channels in the cell membrane, Morikage *et al.* were unable to determine why AmB had effects on the resistant cells, but not on the sensitive parental cell line.

In 1988, Andrews *et al.* observed that the DDP-resistant 2008/C13*5.25 cells, derived from the human ovarian 2008 cell line, accumulated 50% less DDP at one hour than the parental cell line (Andrews *et al.*, 1988). In order to determine if the decreased uptake could be explained by a change in the plasma membrane, Mann *et al.* studied the lipid composition and membrane fluidity of the sensitive and resistant cell lines (Mann *et al.*, 1988). Although there were slight differences in the lipid compositions of the cells, the resultant membrane fluidity was not significantly different in the sensitive and resistant cells when determined by direct measurement of fluorescence polarisation.

To further study the decreased accumulation by the resistant cells, uptake was measured in the presence of metabolic inhibitors (dinitrophenol, sodium fluoride, and iodoacetate) and the sodium-potassium ATPase inhibitor ouabain (Andrews *et al.*, 1988). None of the metabolic inhibitors inhibited DDP uptake when added individually (iodoacetate actually increased uptake, probably through disruption of the cell membrane), but when added in combination, uptake was inhibited by 45%.

The sodium-potassium ATPase inhibitor ouabain inhibited uptake by 25% when cells were exposed to Louabain at a concentration of 0.2 mM for 30 min (Andrews *et al.*, 1988). In a later paper, Andrews *et al.* determined that longer pre-incubation (1 h) with ouabain increased the inhibition of uptake to 50% (Andrews *et al.*, 1991). In order to determine if the effect of ouabain was on the transport of DDP into the cell, and not an effect on metabolism or efflux, short term uptake of $^{195}$Pt-DDP was measured. Ouabain inhibited uptake at time points as early as 1 min. When the ATPase was inactivated by replacing the sodium in the media with choline, or by decreasing the extracellular levels of potassium, DDP uptake was also inhibited by 50% at these early time points. Recently, Andrews *et al.* demonstrated that the sodium/potassium ATPase itself was not directly transporting DDP. By increasing intracellular sodium with the sodium ionophore, monensin, they were able to increase the activity of the ATPase by 160%. However, DDP accumulation was unchanged (Andrews & Albright, 1991). Andrews *et al.* have also demonstrated that DDP accumulation is potassium dependent, and therefore also appears to be dependent on the membrane potential. When the membrane was depolarised by incubation in high potassium media, the cells accumulated 5.4-fold more DDP in 10 min than did the control cells (Andrews & Albright, 1991). They also found that two DDP-resistant sublines, 2008/C13*5.25 and A2780/CP, had elevated membrane potential, suggesting that DDP-accumulation appears to be inversely related to membrane potential. These findings suggest that some component of DDP accumulation is via a process that is dependent upon the cell maintaining an electrochemical gradient across the membrane.

The uptake of DDP has also been shown to be inhibited by the overexpression of the c-Ha-*ras* oncogene (Isonishi *et al.*, 1991). Isonishi *et al.* created an NIH3T3 cell line stabily transfected with the c-Ha-*ras* oncogene gene under the control of the mouse mammary tumour virus promoter. When c-Ha-*ras* expression was induced with dexamethasone, this line exhibited low level resistance to DDP, while dexamethasone had no effect on non-transfected cells. They found that this effect was accompanied by a 40% decrease in DDP accumulation as measured by $^{195}$Pt uptake and an increase in the cellular metallothionein content.

DDP accumulation is also affected by a number of other intracellular signaling mechanisms, including protein kinase C (PKC), protein kinase A (PKA), and the $Ca^{++}$/calmodulin pathway. Mann *et al.* found that cyclic AMP-induced activation of PKA increased the cellular accumulation and toxicity of DDP in drug sensitive human 2008 cells (Mann *et al.*, 1991). This effect was muted in the 2008/C13*5.25 DDP-resistant daughter line. They found that 10 min DDP accumulation could be doubled by treating the cells with 10 μM forskolin and tripled by treating with 10 μM of the phosphodiesterase inhibitor 3-isobutyl-1-methylxanthine (IBMX). Curiously, when 1,9-dideoxyforskolin, an inactive analog of forskolin, was used as a negative control, the accumulation of DDP was decreased. These experiments were repeated in another human ovarian carcinoma cell line, A2780, with the same results, suggesting that the PKA pathway mediates the uptake of DDP.

Howell *et al.* showed that the nucleoside transport inhibitor dipyridamole increased DDP accumulation in 2008 cells in a dose-dependent manner (Howell *et al.*, 1987; Jekunen *et al.*, 1992*b*). Dipyridamole (20 μM) increased DDP accumulation at 1 h by 2-fold. This concentration increased DDP toxicity by 4.3-fold in these cells. Another inhibitor of nucleoside transport, nitrobenzyl thioinosine did not have the same effect, suggesting that inhibition of nucleoside transport was not the mechanism of action. Although dipyridamole inhibits cAMP phosphodiesterase in platelets, subsequent work has shown that this effect was not mediated by increasing intracellular levels of cAMP in 2008 cells (Jekunen *et al.*, 1992*b*).

Isonishi *et al.* demonstrated that phorbol esters were able to sensitise 2008 cells to DDP. However, this effect was not due to increased DDP accumulation (Isonishi *et al.*, 1990). Basu *et al.* confirmed the finding that increased PKC activity sensitised cells to DDP. However, they found that this sensitisation was accompanied by an increase in DDP accumulation. They demonstrated that a 24 h pretreatment of HeLa cells with PKC activators such as TPA or PDBu increased 24 h DDP accumulation by 200% (Basu *et al.*, 1990). This increase in accumulation at 24 h was muted when protein synthesis was inhibited by the addition of cycloheximide. However, cycloheximide had no effect on uptake when the phorbol ester pretreatment was only for an hour. It should be noted that Basu *et al.* pretreated the cells with phorbol esters, while Isonishi *et al.*, treated the cells with phorbol esters and DDP concurrently.

Kikuchi *et al.* studied the effects of blocking the $Ca^{++}$/calmodulin signal transduction pathway using the calmodulin antagonists W-5 and W-7. They observed that the calmodulin antagonists could potentiate the cytotoxicity of DDP in human ovarian carcinoma cells. Measuring DDP accumulation by atomic absorption spectroscopy, it was determined that the mechanism by which the calmodulin antagonists potentiated DDP toxicity was by increasing DDP accumulation (Kikuchi *et al.*, 1990). The uptake of DDP was slightly increased by W-7 in the sensitive parental cell line. In the resistant daughter cell line W-7 doubled the uptake *in vitro* and tripled the uptake into a tumour in nude mice. At present it is unclear how the calmodulin antagonists increase the accumulation of DDP.

### Other modulators of DDP accumulation

There are several things that affect DDP accumulation which do not help discriminate between active transport *vs* passive diffusion of DDP since they are compatible with both theories of DDP transit into the cell. Andrews *et al.* showed that the accumulation of DDP into 2008 cells was inversely dependent on both extracellular pH and osmolality (Andrews *et al.*, 1987). Timmer-Bosscha *et al.* found that docosahexaenoic acid (DCHA) increased the toxicity of DDP in a resistant subline of the human small cell lung carcinoma $GLC_4$, but not in the sensitive parental cell line (Timmer-Bosscha *et al.*, 1989). DCHA, a polyunsaturated fatty acid, was able to increase DDP accumulation 300% at 4 h in the resistant cell line as measured by atomic absorption.

The accumulation of DDP into cells is also temperature dependent. Melvik and Pettersen have shown that NHIK 3025 cells at 22°C are 3-fold resistant to DDP as compared to the same cells at 37°C. Using atomic absorption, they determined that cells at 22°C needed three times the dose of DDP to accumulate the same amount of platinum as they did at 37°C (Melvik & Pettersen, 1988). Mann *et al.* also found that cells at 22°C accumulate 3-fold less DDP than those at 37°C. Using 2008 cells and a resistant subline C13/5.25, they found that accumulation varied linearly with temperature from 12°C to 40°C in both cell lines as determined by atomic absorption (Mann *et al.*, 1988).

DDP accumulation can also be increased by permeabilisation of the plasma membrane. Melvik *et al.* demonstrated that electropermeabilisation of the cell membrane during DDP exposure increased sensitivity to DDP and that this was mediated by a 3-fold increase in DDP accumulation as measured by atomic absorption (Melvik *et al.*, 1986). Jekunen *et al.* demonstrated that uptake could be enhanced by disrupting the cell membrane with heat or digitonin (Jekunen *et al.*, 1992*a*). Exposure to 20 $\mu$M digitonin increased DDP accumulation 2–3-fold over control while exposure to 65°C increased the uptake 4–7-fold.

### Efflux of DDP

Although there have been a number of studies of the uptake and accumulation of DDP, there has been relatively little work reported on the efflux of DDP from cells. This is unfortunate, since Melvik *et al.* reported data which suggests that the plasma membrane is functioning as a barrier against efflux as well as influx. They have shown that electropermeabilisation after DDP exposure protects NHIK 3025 cells from DDP toxicity, presumably by increasing DDP efflux from the cells (Melvik *et al.*, 1992). Mann *et al.* measured the efflux of DDP from 2008 cells by atomic absorption and found that the efflux was biphasic, with a very rapid initial phase followed by a much slower terminal phase (Mann *et al.*, 1990). They also found that the initial efflux was more rapid in the DDP-resistant variant of 2008 cells. Shionoya and Scanlon also found that efflux was biphasic in K562 cells when measured by $^{195}$Pt-DDP retention; the initial efflux was complete in the first 5 min and the remaining DDP was only very slowly effluxable (Shionoya & Scanlon, 1986). Waud observed similar efflux kinetics in L1210 cells as measured by $^{14}$C dichloro(ethylenediammine) platinum retention (Waud, 1987). Neither Shionoya nor Waud found increased efflux in the DDP-resistant variants of their cell lines. Overall the existing data suggests that any differences in efflux between sensitive and resistant cells are of a smaller magnitude than the differences in influx, although it should be emphasised that detailed studies of efflux kinetics of DDP are lacking.

### Working model of DDP accumulation

The current data paint a confusing picture of DDP accumulation in the cell. On the one hand, DDP uptake appears to occur by passive diffusion since it is not saturable nor is it inhibited by structural analogs. On the other hand, the uptake can clearly be modulated both by a variety of pharmacologic agents that do not cause general permeabilisation of the membrane, and by activation of some intracellular signal transduction pathways. An important observation is that in no case has it been reported that uptake can be inhibited by more than 50% of control.

One model that accommodates most of the existing observations envisions that approximately one half of the initial drug uptake rate is due to passive diffusion and that the other half is occurring by facilitated diffusion through a gated channel (Figure 1). In such a system, one would not expect to observe saturation of initial uptake since the rates for both kinds of diffusion are a simple function of external drug concentration. On the other hand, influx through such channels is known to be influenced by many factors. In addition, one would expect the change in accumulation velocity with increasing temperature to be in the intermediate range as has been observed for DDP (Mann *et al.*, 1988). Thus the model can explain each of the major arguments in support of passive diffusion. At the same time, the model accommodates the major arguments in favour of mediated transport. If the channel is lost during selection of drug-resistant variants, then one would expect up to a 50% decrease in the initial rate of DDP uptake, even in the absence of changes in the membrane fluidity. Information on the regulation of a variety of gated channels makes it reasonable to propose that flux through the channel is regulated by phosphorylation cascades initiated by activation of protein kinase A, protein kinase C, or by the calmodulin dependent kinases. The ability of membrane impermeant aldehydes to block 50% of the uptake suggests that critical amino groups of the channel are exposed on the external surface of the cell. The fact that ouabain inhibits 50% of the initial uptake suggests that ion gradients maintained by this ATPase are crucial to the function of this channel. This hypothesis is further strengthened by the fact that proper function of the channel is dependent upon maintenance of the proper membrane potential. The model also provides a reasonable explanation for why so many DDP-resistant sublines exhibit decreased DDP accumulation as a phenotype. A single base pair mutation in the transmembrane region of such a protein would be sufficient to inhibit the channel's function. Since DDP is a reasonably good mutagen in mammalian cells, the DDP selection itself could cause a loss of function mutation. Conversely, a mutation that hyperpolarises the cell, could keep the channel in the closed confirmation.

**Figure 1** A working model of DDP accumulation. DDP can enter the cell either by way of passive diffusion or through a gated channel. The flux through this channel can be increased by the agents on the right: docosahexaenoic acid (DCHA), dipyridamole, 1-propargyl-5-chloropyrimidin-2-one (NY 3170), calmodulin inhibitors (W-7), amphotericin B (AmB), and phosphorylation by either PKA or PKC. The flux through the channel can be decreased by various aldehydes. The flux through this channel is also dependent on a functional $Na^{+}/K^{+}$ ATPase, membrane potential, extracellular pH and extracellular osmolality.

**Table I** Agents that modulate DDP accumulation

| *Agents that increase accumulation* | *Agents that decrease accumulation* |
|---|---|
| 1-propargyl-5-chloropyrimidin-2-one (Dornish *et al.*, 1987) | Aldehydes (Dornish *et al.*, 1986; Dornish *et al.*, 1989) |
| Amphotericin B (Morikage *et al.*, 1991) | Decreased intracellular ATP (Andrews *et al.*, 1988) |
| PKA activators (Mann *et al.*, 1991) | Ouabain (Andrews *et al.*, 1988) |
| PKC activators (Basu *et al.*,1990) | Low extracellular sodium (Andrews *et al.*, 1988) |
| Dipyridamole (Howell *et al.*, 1987; Jekunen *et al.*, 1992*b*) | c-Ha-*ras* (Isonishi *et al.*, 1991) |
| Calmodulin antagonists (Kirkuchi *et al.*, 1990) | Low extracellular potassium (Andrews *et al.*, 1991) |
| Low extracellular pH (Andrews *et al.*, 1987) | |
| Low extracellular osmolality (Andrews *et al.*, 1987) | |
| High extracellular potassium (Andrews & Albright, 1991) | |
| Docosahexaenoic Acid (Timmer-Bosscha *et al.*, 1989) | |

Based on this model, one would predict that analogs of cisplatin that are substantially more lipophilic would not exhibit decreased uptake in resistant cells and would therefore have only limited cross-resistance. Presumably, such lipophilic analogs would be less dependent on the gated channel for entry into the cell. Kraker and Moore have observed that in some DDP-resistant L1210 cells, there is no cross-resistance to DACH-DDP (Kraker & Moore, 1988). However, Nicolson *et al.* have shown considerable cross-resistance in other L1210-derived resistant cells (Nicolson *et al.*, 1992). Using the more lipophilic ammine/amine platinum (IV) dicarboxylates, Kelland *et al.* have been able to overcome resistance in accumulation defective human 41McisR cells. These drugs could not overcome resistance in the CH1cisR cell line that had normal DDP accumulation. Although they did not measure platinum accumulation in this study, it is probable that these drugs were able to overcome the accumulation defect in the 41McisR cells (Kelland *et al.*, 1992).

If wild-type cells contain a channel protein, and the amount of this protein is altered in resistant cells with decreased accumulation, then one might expect to be able to develop antibodies differentially reactive with membrane proteins of sensitive and resistant cells. In fact, Kawai *et al.* have developed a rabbit polyclonal antibody raised against a DDP-resistant murine lymphoma cell line which detects a 200 kD protein that is overexpressed in the resistant cells as compared to the parental cell line (Kawai *et al.*, 1990). The amount of this 200 kD protein was inversely related to the DDP accumulation in these cell lines. They have postulated that this protein is analogous to p-glycoprotein. However it is equally possible that this protein represents a non-functional gated channel produced by the cell in excess amounts in an effort to compensate for the loss of the channel. Bernal *et al.* have developed a mouse monoclonal antibody (SQM1) against a human squamous cell carcinoma that is decreased in DDP and methotrexate resistant cells (Bernal *et al.*, 1991). The drug resistant variant expresses only 20% of the SQM1 protein detectable in the parental cell line as quantitated by indirect radioimmunoassay. Such a decrease in SQM1 has been confirmed in the human small cell carcinoma, SW2-S. The gene encoding SQM1 has been cloned and predicts a protein of 135 amino acids. This protein does not have a recognisable transmembrane region, however it does appear to have a secretory leader sequence. Bernal *et al.* have postulated that SQM1 is an extracellular protein that interacts with membrane spanning proteins to facilitate DDP and methotrexate transport into the cells.

In conclusion, DDP enters the cell through a process which although not saturable, is able to be modulated by a variety of agents. While the discovery that one can modulate uptake is leading to the development of new therapeutic strategies, since decreased accumulation is a common feature of DDP resistance, additional information on the mechanisms of uptake is urgently needed. Although the cellular control of DDP accumulation is undoubtedly more complicated than described in this model, and quite probably varies in different cell lines, the major virtue of this model is that it permits the design of specific experiments to further define the mechanisms of DDP entry into cells.

This work was supported in part by grant CA-377 from the American Cancer Society, and grant 100R47 from Bristol-Myers Squibb. This work was conducted in part by the Clayton Foundation for Research -California Division. Dr Howell is a Clayton Foundation Investigator.

Abbreviations: DMSO, dimethyl sulfoxide; DACH-Pt, 1,2-diaminocyclohexaneplatinum sulfate; cAMP, cyclic adenosine monophosphate; TPA, 12-O-tetradecanoylphorbol 13-acetate; PDBu, phorbol 12,13-dibutyrate.

### References

ANDREWS, P.A. (1991). Lack of inhibition of DDP uptake by platinum analogs. (Personal communication).

ANDREWS, P.A. & ALBRIGHT, K.A. (1991). Role of membrane ion transport in cisplatin accumulation. In *Platinum and other metal coordination compounds in cancer chemotherapy*, Howell, S.B. (ed). pp. 151–159. Plenum Press: New York, NY.

ANDREWS, P.A. & HOWELL, S.B. (1990). Cellular pharmacology of cisplatin: perspectives on mechanisms of acquired resistance. *Cancer Cells*, **2**, 35–43.

ANDREWS, P.A., JONES, J.A., VARKI, N.M. & HOWELL, S.B. (1990). Rapid emergence of acquired *cis*-diamminedichloroplatinum(II) resistance in an *in vivo* model of human ovarian carcinoma. *Cancer Comm.*, **2**, 93–100.

ANDREWS, P.A., MANN, S.C., HUYNH, H.H. & ALBRIGHT, K.D. (1991). Role of the $Na^+$, $K^+$-adenosine triphosphatase in the accumulation of *cis*-diamminedichloroplatinum(II) in human ovarian carcinoma cells. *Cancer Res.*, **51**, 3677–3681.

ANDREWS, P.A., MANN, S.C., VELURY, S. & HOWELL, S.B. (1987). Cisplatin uptake mediated cisplatin-resistance in human ovarian carcinoma cells. In *Platinum and other metal coordination compounds in cancer chemotherapy*, Nicolini, M. (ed.). pp. 248-254. Martinus Nijhoff Publishing: Padua, Italy.

ANDREWS, P.A., VELURY, S., MANN, S.C. & HOWELL, S.B. (1988). *cis*-diamminedichloroplatinum(II) accumulation in sensitive and resistant human ovarian carcinoma cells. *Cancer Res.*, **48**, 68–73.

BASU, A., TEICHER, B.A. & LAZO, J.S. (1990). Involvement of protein kinase C in phorbol ester-induced sensitization of HeLa cells to *cis*-diamminedichloroplatinum(II). *J. Biol. Chem.*, **265**, 8451–8457.

BERNAL, S.D., WONG, Y.-C., KAKEFUDA, M. & URBANO, A.G. (1991). A new membrane protein associated with resistance to cis-platinum and methotrexate. In *Proceeding of the sixth international symposium on platinum and other metal coordination compounds in cancer chemotherapy*, Howell, S.B. (ed.). pp. 323-334. Plenum Press: San Diego, CA.

BINKS, S.P. & DOBROTA, M. (1990). Kinetics and mechanics of uptake of platinum-based pharmaceuticals by the rat small intestine. *Biochem. Pharmacol*, **40**, 1329–1336.

BYFIELD, J.E. & CALABRO-JONES, P.M. (1981). Carrier-dependent and carrier-independent transport of anti-cancer alkylating drugs. *Nature*, **294**, 281–283.

DORNISH, J.M., MELVIK, J.E. & PETTERSEN, E.O. (1986). Reduced cellular uptake of *cis*-dichlorodiammineplatinum(II) by benzaldehyde. *Anticancer Res.*, **6**, 583–588.

DORNISH, J.M. & PETTERSEN, E.O. (1985). Protection from *cis*-dichlorodiammineplatinum-induced cell inactivation by aldehydes involves cell membrane amino groups. *Cancer Letters*, **29**, 235–243.

DORNISH, J.M., PETTERSEN, E.O. & OFTEBRO, R. (1987). Synergistic cell inactivation by cis-dichlorodiammineplatinum(II) in combination with 1-propargyl-5-chloropyrimidin-2-one. *Br. J. Cancer*, **56**, 273–278.

DORNISH, J.M., PETTERSEN, E.O. & OFTEBRO, R. (1989). Modifying effect of cinnamaldehyde and cinnamaldehyde derivatives on cell inactivation and cellular uptake of *cis*-diamminedichloroplatinum (II) in human NHIK 3025 cells. *Cancer Res.*, **49**, 3917–3921.

DORNISH, J.M., PETTERSEN, E.O., OFTEBRO, R. & MELVIK, J.E. (1984). Reduction of *cis*-dichlorodiammineplatinum(II)-induced cell inactivation by benzaldehyde. *Eur. J. Clin. Oncol.*, **20**, 1287–1293.

GALE, G.R., MORRIS, C.R., ATKINS, L.M. & SMITH, A.B. (1973). Binding of an antitumor platinum compound to cells as influenced by physical factors and pharmacologically active agents. *Cancer Res.*, **33**, 813–818.

HOWELL, S.B., VICK, J., ANDREWS, P.A., VELURY, S. & SANGA, R. (1987). Biochemical modulation of cisplatin. In *Platinum and other metal coordination compounds in cancer chemotherapy*, Nicolini, M. (ed.). pp. 228–234. Martinus Nijhoff Publishing: Padua, Italy.

HROMAS, R.A., NORTH, J.A. & BURNS, C.P. (1987). Decreased cisplatin uptake by resistant L1210 leukemia cells. *Cancer Letters*, **36**, 197–201.

ISONISHI, S., ANDREWS, P.A. & HOWELL, S.B. (1990). Increased sensitivity to *cis*-diamminedichloroplatinum(II) in human ovarian carcinoma cells in response to treatment with 12-*O*-tetradecanoylphorbol 13-acetate. *J. Biol. Chem.*, **265**, 3623–3627.

ISONISHI, S., HOM, D.K., THIEBAUT, F.B., MANN, S.C., ANDREWS, P.A., BASU, A., LAZO, J.S., EASTMAN, A. & HOWELL, S.B. (1991). Expression of the c-Ha-*ras* oncogene in mouse NIH 3T3 cells induces resitance to cisplatin. *Cancer Res.*, **51**, 5903–5909.

JEKUNEN, A.J., SHALINSKY, D.R., HEATH, D.D., KHATIBI, S., ALBRIGHT, K.D. & HOWELL, S.B. (1992*a*). Modulation of cisplatin cytotoxicity by permeabilization of the plasma membrane using digitonin *in vitro* and *in vivo*. In *Proceedings of the Eighty-Third Annual Meeting of the American Association for Cancer Research*, (ed.). pp. 419. : San Diego, CA.

JEKUNEN, A.J., VICK, J., SANGA, R., CHAN, T.C.K. & HOWELL, S.B. (1992*b*). Synergism between dipyridamole and cisplatin in human ovarian carcinoma cells *in vitro*. *Cancer Res.*, (in press).

KAWAI, K., KAMATANI, N., GEORGES, E. & LING, V. (1990). Identification of a membrane glycoprotein overexpressed in murine lymphoma sublines resistant to *cis*-diamminedichloroplatinum(II). *J. Biol. Chem.*, **265**, 13137–13142.

KELLAND, L.R., MISTRY, P., ABEL, G., LOH, S.Y., O'NEILL, C.F., MURRER, B.A. & HARRAP, K.R. (1992). Mechanism-related circumvention of acquired *cis*-diamminedichloroplatinum(II) resistance using two pairs of human ovarian carcinoma cell lines by ammine/amine platinum(IV) dicarboxylates. *Cancer Res.*, **52**, 3857–3864.

KELLEY, S.L. & ROZENCWEIG, M. (1989). Resistance to platinum compounds: mechanisms and beyond. *Eur. J. Clin. Oncol.*, **25**, 1135–1140.

KIKUCHI, Y., IWANO, I., MIYAUCHI, M., SASA, H., NAGATA, I. & KUKI, E. (1990). Restorative effects of calmodulin antagonists on reduced cisplatin uptake by cisplatin-resistant human ovarian carcinoma cells. *Gynecol Oncol.*, **39**, 199–203.

KRAKER, A.J. & MOORE, C.W. (1988). Accumulaton of *cis*-diamminedichloroplatinum(II) and platinum analogues by platinum-resistant murine leukemia cells *in vitro*. *Cancer Res.*, **48**, 9–13.

MANN, S.C., ANDREWS, P.A. & HOWELL, S.B. (1988). Comparison of lipid content, surface membrane fluidity, and temperature dependence of *cis*-diamminedichloroplatinum(II) accumulation in sensitive and resistant human ovarian carcinoma cells. *Anticancer Res.*, **8**, 1211–1216.

MANN, S.C., ANDREWS, P.A. & HOWELL, S.B. (1990). Short-term cis-diamminedichloroplatinum(II) accumulation in sensitive and resistant human ovarian carcinoma cells. *Cancer Chemother. Pharmacol*, **25**, 236–240.

MANN, S.C., ANDREWS, P.A. & HOWELL, S.B. (1991). Modulation of *cis*-diamminedichloroplatinum(II) accumulation and sensitivity by forskolin and 3-isobutyl-1-methylxanthine in sensitive and resistant human ovarian carcinoma cells. *Int. J. Cancer*, **48**, 866–872.

MELVIK, J.E., DORNISH, J.M. & PETTERSEN, E.O. (1992). The binding of *cis*-dichlorodiammineplatinum(II) to extracellular and intracellular compounds in relation to drug uptake and cytotoxicity *in vitro*. *Br. J. Cancer*, **66**, 260–265.

MELVIK, J.E. & PETTERSEN, E.O. (1988). Oxygen and temperature-dependent cytotoxicity and radiosensitizing effects of *cis*-dichlorodiammineplatinum(II) on human NHIK 3025 cells *in vitro*. *Radiation Res.*, **114**, 489–499.

MELVIK, J.E., PETTERSEN, E.O., GORDON, P.B. & SEGLEN, P.O. (1986). Increase in *cis*-dichlorodiammineplatinum(II) cytotoxicity upon reversible electropermeabilization of the plasma membrane in cultured human NHIK 3025 cells. *Eur. J. Clin. Oncol.*, **22**, 1523–1530.

MORIKAGE, T., BUNGO, M., INOMATA, M., YOSHIDA, M., OHMORI, T., FUJIWARA, Y., NISHIO, K. & SAIJO, N. (1991). Reversal of cisplatin resistance with Amphotericin B in non-small cell lung cancer cell line. *Jpn. J. Cancer Res.*, **82**, 747–751.

NICOLSON, M.C., ORR, R.M., O'NEILL, C.F. & HARRAP, K.R. (1992). The role of platinum uptake and glutathione levels in L1210 cells sensitive and resistant to cisplatin, tetraplatin, or carboplatin. *Neoplasma*, **39**, 189–195.

OGAWA, M., GALE, G.R. & KEIRN, S.S. (1975). Effects of *cis*-diamminedichloroplatinum (NSC 119875) on murine and human hemopoietic precursor cells. *Cancer Res.*, **35**, 1398–1401.

PEREZ, R.P., HAMILTON, T.C. & OZOLS, R.F. (1990). Resistance to alkylating agents and cisplatin: insights from ovarian carcinoma model systems. *Pharmacol. Ther.*, **48**, 19–27.

SHIONOYA, S. & SCANLON, K.J. (1986). Properties of amino acid transport systems in K562 cells sensitive and resistant to *cis*-diamminedichloroplatinum(II). *Cancer Res.*, **46**, 3445–3448.

TIMMER-BOSSCHA, H., HOSPERS, G.A.P., MEIJER, C., MULDER, N.H., MUSKIET, F.A.J., MARTINI, I.A., UGES, D.R.A. & DE VRIES, E.G.E. (1989). Influence of docosahexaenoic acid on cisplatin resistance in a small cell lung carcinoma cell line. *J. Natl Cancer Inst.*, **81**, 1069–1075.

TIMMER-BOSSCHA, H., MULDER, N.H. & DE VRIES, E.G.E. (1992). Modulation of *cis*-diamminedichloroplatinum(II) resistance: a review. *Br. J. Cancer*, **66**, 227–238.

WAUD, W.R. (1987). Differential uptake of *cis*-diamminedichloroplatinum(II) by sensitive and resistant murine L1210 leukemia cells. *Cancer Res.*, **47**, 6549–6555.